# Small Sample Stress: Probing Oxygen-Deprived Ammonia-Oxidizing Bacteria with Raman Spectroscopy In Vivo

**DOI:** 10.3390/microorganisms8030432

**Published:** 2020-03-19

**Authors:** Ann-Kathrin Kniggendorf, Regina Nogueira, Somayeh Nasiri Bahmanabad, Andreas Pommerening-Röser, Bernhard Wilhelm Roth

**Affiliations:** 1 Hannover Centre for Optical Technologies, Gottfried Wilhelm Leibniz University of Hannover, Nienburger Str. 17, 30167 Hannover, Germany; 2 Institute for Sanitary Engineering and Waste Management, Gottfried Wilhelm Leibniz Universität Hannover, Welfengarten 1, 30167 Hannover, Germany; 3 Microbiology and Biotechnology, University of Hamburg, Ohnhorststr. 18, 22609 Hamburg, Germany; 4 Cluster of Excellence PhoenixD (Photonics, Optics, and Engineering – Innovation Across Disciplines), Welfengarten 1, 30167 Hannover, Germany

**Keywords:** confocal Raman microscopy, *Nitrosomonas*, *Nitrosospira*, oxygen deprivation, cytochrome c, resonance Raman spectroscopy

## Abstract

The stress response of ammonia-oxidizing bacteria (AOB) to oxygen deprivation limits AOB growth and leads to different nitrification pathways that cause the release of greenhouse gases. Measuring the stress response of AOB has proven to be a challenge due to the low growth rates of stressed AOB, making the sample volumes required to monitor the internal stress response of AOB prohibitive to repeated analysis. In a proof-of-concept study, confocal Raman microscopy with excitation resonant to the heme c moiety of cytochrome c was used to compare the cytochrome c content and activity of stressed and unstressed *Nitrosomonas europaea* (Nm 50), *Nitrosomonas eutropha* (Nm 57), *Nitrosospira briensis* (Nsp 10), and *Nitrosospira* sp. (Nsp 02) in vivo. Each analysis required no more than 1000 individual cells per sampling; thus, the monitoring of cultures with low cell concentrations was possible. The identified spectral marker delivered reproducible results within the signal-to-noise ratio of the underlying Raman spectra. Cytochrome c content was found to be elevated in oxygen-deprived and previously oxygen-deprived samples. In addition, cells with predominantly ferrous cytochrome c content were found in deprived *Nitrosomonas eutropha* and *Nitrosospira* samples, which may be indicative of ongoing electron storage at the time of measurement.

## 1. Introduction

Ammonia-oxidizing bacteria (AOB), along with ammonia-oxidizing archaea, commammox, and nitrite-oxidizing bacteria, are key players in nitrogen cycling both in natural and engineered water environments and in the soil [1,2]. AOB are affiliated to the *β* and *γ* subclasses within the Proteobacteria, with the majority of species belonging to the *β* subclass [3,4]. AOB within the *β* subclass are divided into the *Nitrosomonas* group and the *Nitrosospira* lineage [3]. Within the *Nitrosomonas* group, *N. europaea* and *N. eutropha* prefer habitats in wastewater treatment plants and eutrophic freshwater. Both species are characterized by a relatively high ammonia tolerance (400 mM and 600 mM for *N. europaea* and *N. eutropha*, respectively), and are halotolerant or moderately halophilic [5]. Species of the genus *Nitrosospira* occur commonly in soils and have been occasionally found in soils and freshwater environments. The maximum ammonia tolerance of *Nitrosospira briensis*, 200 mM, is lower than that of *N. europaea* or *N. eutropha*. Salt requirements have not been described for *Nitrosospira.* [6].

In engineered and natural environments, AOB constantly face stress conditions such as ammonia limitation and starvation, low dissolved oxygen concentration, low temperature, etc. *N. europaea* survives periods of ammonia starvation well, regaining its ammonia-oxidizing activity swiftly after supplementation of ammonium [7,8]. Microbial nitrification, i.e., the metabolization of ammonia to nitrite, performed by AOB under aerobic conditions is crucial for the biological treatment of wastewater [2]. However, in cases of oxygen limitation, additional nitrogenous gases such as nitric oxide (NO) and nitrous oxide (NO_2_) are produced [6,9,10]. In soil management, this negatively affects the efficiency of fertilizer, leading to nitrogen loss for the crops, an increased release of greenhouse gases from the soil, and eutrophication of water bodies downstream [11,12,13]. Measuring the stress response of the present AOB that are leading to different nitrification pathways is therefore a crucial task, especially since prediction of the AOB response has proven to be a challenge. Predicting the stress response of AOB to different dissolved oxygen concentrations has even been deemed impossible [14]. 

**Figure 1:**
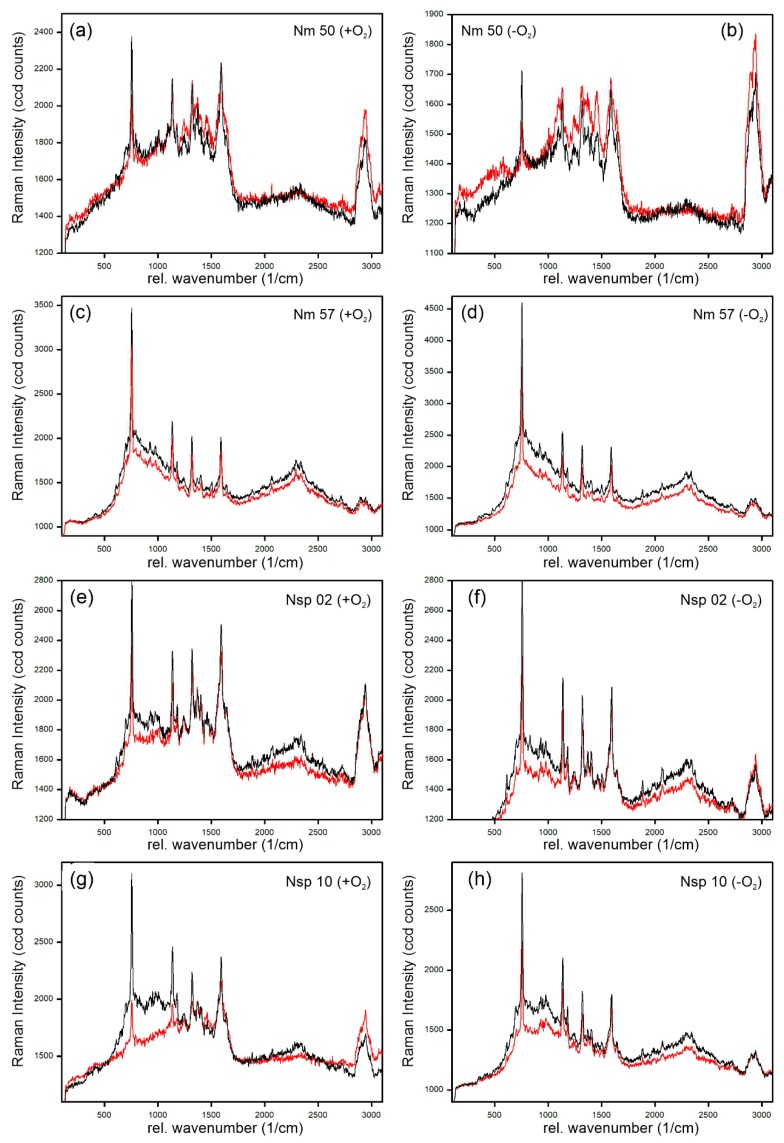
Cytochrome-c-resonant Raman spectra of *Nitrosomonas europaea* (Nm 50) (**a**,**b**), *Nitrosomonas eutropha* (Nm 57) (**c**,**d**), *Nitrosospira* sp. (Nsp 02) (**e**,**f**), and *Nitrosospira briensis* (Nsp 10) (**g**,**h**) as recorded in vivo from individual bacteria cells in well-aerated (**a**,**c**,**e**,**g**) and oxygen-deprived (**b**,**d**,**f**,**h**) cultures. Black spectra represent a maximal relRI (most ferrous cytochrome c content of the cell), and red spectra a minimal relRI (least ferrous) for the respective culture. Please note the different intensity ranges.

Recently, the response of AOB to either ammonia or dissolved oxygen deprivation has been studied extensively based on transcriptomics [9,15], proteomics [16], and genome analysis [10], finding that *N. europaea*, *N. eutropha*, and *Nitrosospira* all encode multiple copies of ammonia monooxygenase (AMO), hydroxylamine dehydrogenase (HAO), and nitride oxide reductase (NOR) as central components of their ammonia oxidation pathways. The electron transport along these pathways is predominantly performed by several cytochrome c proteins, some of which hold more than one heme c in their protic matrix [9,10,17,18,19,20,21]. For a scheme of the electron transport and the ammonia oxidation pathways for *N. europaea*, including the associated cytochrome c proteins, see Sedlacek et al. [9] (Figure 1). 

A comparative quantitative proteomic study of growing and ammonia-starved *N. europaea* cells by Pellitteri-Hahn et al. [16] found that energy-starved cells shifted their cellular activity from biosynthesis toward cellular survival functions, reporting a significantly increased abundance of cytochrome c in the ammonia-starved cells as compared to growing cells, which was interpreted as a cellular strategy for a rapid growth response. However, by comparing transcript levels of growing cells and ammonia-deprived *N. europaea* cells, Wei et al. [22] reported in contrast that the number of mRNAs detected and their overall amounts were greater during exponential growth than during ammonia deprivation. The mRNA levels for cytochrome c reported by Wei et al. were more than 10-fold higher in growing cells relative to ammonia deprived cells. 

Yu and Chandran [15] reported for *N. europaea* batch cultures an increase in exponential phase mRNA concentrations of both AMO and HAO genes with decreasing dissolved oxygen concentrations, suggesting a mechanism to metabolize ammonia and hydroxylamine more effectively under dissolved oxygen limitation. Sedlacek et al. [9] investigated the overall effect of oxygen limitation on *N. europaea* using steady-state cultivation with whole-genome transcriptomics. They reported that the heme–copper-containing cytochrome-c oxidases encoded by *N. europaea* were upregulated during oxygen-limited growth.

In the case of *Nitrosospira briensis*, a decrease in both ammonia-oxidizing activity and AMO mRNA expression was observed during a two-week starvation period, while the opposite happened during resuscitation [23]. The patterns of the soluble protein fraction of *N. briensis* culture revealed only small changes between growing and ammonium-starved cells.

However, all the methods employed in the aforementioned studies are destructive by nature and require comparatively large sample volumes, and thus are self-limiting for observing cultures with low cell growth due to oxygen or ammonia deprivation. 

In contrast, confocal Raman microscopy is a powerful tool for the analysis of microbial cells and communities in vivo and in situ, even providing optical access to the proteome [24,25,26]. In combination with multivariate data analysis [27,28,29], it has proven suitable for the in vivo discrimination of microbial species at the cell level. For example, Maquelin et al. used confocal Raman microscopy with 830 nm excitation to discriminate several strains of *Acinetobacter* in dried samples [30], while Dina et al. used confocal Raman microscopy with 785 nm excitation and fuzzy principal component analysis to discriminate three halo-archaeal genera in vivo [29]. Incidentally, we used confocal Raman microscopy with cytochrome-c-resonant excitation at 532 nm and hierarchical cluster analysis for the in vivo discrimination and identification of several species of purple non-sulfur bacteria and AOB in planktonic cultures, as well as associated in microbial wastewater biofilms [28,31,32]. 

While the crucial enzymes AMO, HAO, and NOR of the ammonia oxidation pathways were not directly accessible with Raman microscopy in vivo, it was possible to target the cytochrome c responsible for the required electron transport along these pathways with resonance Raman micro-spectroscopy, a technique also uniquely positioned to determine conformational changes of cytochrome c and its redox state (ferrous vs. ferric hemes c). Hu et al. [33], in their annotation of the resonance Raman spectra of extracted cytochrome c, previously reported large changes in both the resonant excitation wavelength and the specifically enhanced molecular vibrations dependent on the redox state of the protein. The most notable change was in the symmetric pyrrole ring breathing mode (B_1g_ in the porphyrin mode assignment according to Li et al. [34]) ν_15_ at 750 rel. cm^−1^, which at 532 nm excitation is one of the strongest Raman lines in ferrous cytochrome c, but is very weak if the protein is in ferric state. Recently, Russo et al. used Raman microscopy with cytochrome-c-resonant excitation at 532 nm, using the redox state of cytochrome c as a crucial indicator for cell apoptosis [35], while Kakita et al. used 532 nm excitation to quantify the redox state of cytochromes b and c in yeast [36]. Shin et al. monitored photothermally induced mitochondrially mediated apoptosis in situ based on the Raman spectrum of cytochrome c, using surface-enhanced resonant Raman microscopy with gold nanoparticles and 785 nm excitation [37]. A similar approach with 633 nm excitation was used by Zhang et al. for the quantitative detection of cytochrome c in living cells [38], and Zhu et al. studied the influence of redox state on cytochrome c release in apoptosis using Raman microscopy with nickel substrates [39].

In this work, we present a proof of concept of the use of straightforward confocal Raman microscopy with 532 nm excitation, resonant to the Q-band of the heme c moiety in cytochrome c, to non-invasively and in vivo analyze the response of four species of AOB, *Nitrosomonas europaea*, *Nitrosomonas eutropha*, *Nitrosospira briensis*, and *Nitrosospira* sp. to oxygen deprivation in comparison to non-oxygen-deprived cultures based on 1000 individual cells per sampling, confirming elevated cytochrome c content and activity following oxygen deprivation in a purely optical measurement with low impact on culture volumes.

## 2. Materials and Methods 

### 2.1. Bacterial Cultures

The strains of ammonia-oxidizing bacteria used in this study were *Nitrosomonas europaea* (Nm 50)*, Nitrosomonas eutropha* (Nm 57)*, Nitrosospira briensis* (Nsp 10), and *Nitrosospira* sp. (Nsp 02). Stock liquid cultures were provided by Dr. Andreas Pommerening-Röser from the University of Hamburg, Germany.

### 2.2. Culture Conditions

The strains of ammonia-oxidizing bacteria Nm 50, Nm 57, Nsp 2, and Nsp 10 were grown in conical flasks (300 mL) at 28 °C in 150 mL basalt mineral salt medium containing 10 mM NH_4_Cl, 0.4 mM KH_2_PO_4_, 1 mM KCl, 0.2 mM MgSO_4_·7H_2_O, 1 mM CaCl_2_·2H_2_O, 10 mM NaCl, 1 mL 0.05 % cresol red solution, and 1 mL trace elements solution (containing per liter: 0.25 N HCl, 0.02 mM MnSO_4_, 0.8 mM H_3_BO_3_, 0.15 mM ZnSO_4_, 0.03 mM (NH_4_)_6_Mo_7_O_24_, 3.5 mM FeSO_4_, and 0.1 mM CuSO_4_). Reagents were purchased from Merck KGaA (Darmstadt, Germany). The medium was prepared according to Krümmel and Harms [40].

The strains of ammonia-oxidizing bacteria were incubated with aeration (shaking at 120 rpm) and without shaking to obtain different concentrations of dissolved oxygen in the medium (MaxQ^TM^ 445 Orbital Shaker, Thermo Fisher Scientific GmbH, Dreieich, Germany). The incubation time was dependent on the growth rate of the respective strain under the prevailing oxygen concentration: 14 d for Nm 50 with shaking, 13 d for Nm 50 without shaking, 11 d for Nm 57 with shaking, 17 d for Nm 50 without shaking, 17 d for Nsp 10 with and without shaking, and 18 d for Nsp 2 with and without shaking. Bacteria were collected for subsequent Raman analysis when the nitrite concentration in the medium was in the range of 7.5 mM to 9.5 mM. During incubation with shaking, the dissolved oxygen concentration was always above 6 mg/L. This situation is referred hereinafter as the well-aerated (+O_2_) growth condition. A sharp decrease in oxygen concentration was observed during incubation without shaking in the first days, followed by a period with lower oxygen concentration. This situation is referred to hereinafter as the oxygen-deprived (−O_2_) growth condition. The formation of nitrite was measured by the method of Bremner [41]. The oxygen concentration in the culture medium was measured using optical oxygen sensor spots attached to the inside glass wall of the conical flasks and completely immersed in the medium. The signal was transferred to a fiber optic trace oxygen meter Fibox 4 (PreSens-Precision Sensing GmbH, Regensburg, Germany) via a fiber optic cable. Cells were harvested in the late growth phase via centrifugation (8000 *g* for 1 h) and were immediately used in Raman spectroscopy.

### 2.3. Resonance Raman Measurements

Bacteria cell solution was deposited on an indentation slide and sealed with a 0.17 mm cover slip. The prepared sample was placed under the microscope and cells were allowed to settle for 15 min before the Raman spectra were recorded.

Raman measurements were performed at room temperature with a confocal Raman microscope (CRM200 by WITec GmbH, Ulm, Germany) equipped with a water-immersion objective (Nikon CFI Fluor by Nikon Instruments Europe B.V., Amsterdam, Netherlands) with a magnification of 60× and a Numerical Aperture NA of 1.0. A frequency-doubled continuous-wave Nd:YAG laser at 531.9 nm, suitable for resonantly enhancing the Q-band of the heme c moiety of cytochrome c in its ferrous (Fe^2+^) state, was used for excitation. Photons from Rayleigh scattering were blocked with a notch filter, covering the spectral range from −120 to 120 rel. cm^−1^. The system had an ellipsoid measurement volume of approx. 1 µm³ defined by the lens properties (assumed refraction index within the sample: 1.33 (water)). The spatial resolution in the horizontal plane was 350 nm, and 2.0 µm perpendicular to it. The spectrometer slit width was 50 µm, realized by a multimode fiber connecting the Raman microscope with the spectrometer (UHTS 300 by WITec GmbH, Ulm, Germany). The grating used had 600 lines per millimeter. Spectra were recorded with an electron charge-coupled device (emCCD) camera (ANDOR DU970N-BV-353 by Andor Technologies Ltd., Belfast, UK), electrically cooled to −69 °C. The spectral resolution of the setup was 4 cm^−1^, with a spectral accuracy of 2 cm^−1^. The recorded spectra covered the range between −80 and 3710 rel. cm^−1^. The laser intensity was adjusted to 36 mW. The loss within the optics prior to the sample contact was 30 per cent. Measurement time per spectrum was set to 1.5 s if not stated otherwise. 

Heme-c-resonant Raman spectra were recorded from individual living cells in a single layer on the bottom of the indentation slide. Measured cells had a minimal distance of 3 µm from one another to avoid effects of photo-bleaching or thermal damage. Control against photo-bleaching or thermal damage was done using white light microscopy before and after the Raman spectra recording, and by repeating the Raman measurement of the cells after a recovery time of two hours.

### 2.4. Data Preparation and Spectral Analysis

Since cytochrome-c-resonant Raman spectra always contain a fluorescent background originating from the cytochrome itself, the Raman spectra were baseline corrected using the IMM algorithm developed by Koch et al. [42], which combines computational efficiency with reliable maintaining of line shapes and relative intensities against the background of strong fluorescence often found in Raman spectra recorded in vivo [43,44]. Spectrum quality was assessed by calculating the signal-to-noise ratio (SNR) for the four dominant resonant Raman lines at 750 (ν_15_), 1130 (ν_22_), 1314 (ν_21_), and 1587 (ν_19_) rel. cm^−1^. Only background-corrected Raman spectra with a SNR > 2 for all four Raman lines were used in the subsequent analysis, which consisted of calculating the relative intensity of the Raman lines at 750 rel. cm^−1^ and 1587 rel. cm^−1^ (relRI) for each spectrum. The calculated relRI of all analyzed cells for each sample were sorted in an ascending order to display the trends for the respective samples. All analysis computations were done in MATLAB [45].

## 3. Results and Discussion

### 3.1. Cytochrome-C-Resonant Raman Spectra and Relative Raman Line Intensity (relRI)

Examples of cytochrome-c-resonant Raman spectra as recorded from native cultures of *Nitrosomonas europaea* (Nm 50), *Nitrosomonas eutropha* (Nm 57), *Nitrosospira briensis* (Nsp 10), and *Nitrosospira* sp. (Nsp 02) under well aerated (+O_2_) and oxygen-deprived (−O_2_) growth conditions are shown in Figure 1. The Raman lines of the four main vibrations of resonantly excited cytochrome c, ν_15_, ν_22_, ν_21_, and ν_19_ at 750, 1130, 1314, and 1587 rel. cm^−1^, respectively, were clearly visible in all spectra, independent of culture and growth conditions. No line shift was observed for these four Raman lines in the spectra, independent of culture and growth conditions. However, significant differences in the relative intensity of the Raman line at 750 rel. cm^−1^ (ν_15_) compared to the line at 1587 rel. cm^−1^ (ν_19_) did exist in the spectra of individual cells per culture (compare red and black spectra in Figure 1a–h). 

This fit perfectly with the results of Hu et al. [33], who analyzed reconstituted cytochrome c protein in potassium phosphate buffer solution, identifying the lines in the complete resonance Raman spectra of ferrous and ferric cytochrome c. With 532 nm excitation, they found the Raman line of the ν_15_ vibration at 750 rel. cm^−1^ to be among the strongest lines in the spectrum of ferrous cytochrome c, but among the weakest lines in ferric cytochrome c, whereas the Raman line of the ν_19_ vibration at 1587 rel. cm^−1^ was unaffected by the redox state of the protein in terms of both relative intensity and line position. Moreover, no line shift of the Raman line at 1587 rel. cm^−1^ was observed. In contrast, Russo et al. observed a 5 cm^−1^ upshift in the line at 1587 rel. cm^−1^ and used it to differentiate between healthy and apoptotic and pre-apoptotic brain cells of Mongolian gerbils, associating the line shift with cytochrome c deformation due to the protein binding to membrane elements [35]. A similar observation was reported earlier by Murgida and Hildebrandt [46,47], who saw a 10 cm^−1^ upshift in the line of the ν_19_ vibration in ferrous compared to ferric cytochrome c adsorbed to a surface-enhanced Raman active substrate. The absence of such a shift or a broadening of the line at 1587 rel. cm^−1^, which would be caused by such a shift in the spectra of some but not all the cytochrome c within the respective cell, therefore indicated that the observed differences in the summarized cytochrome c resonant Raman spectra of the bacteria were indeed primarily due to the redox state of the cytochrome c, as analyzed by Hu et al. [33], and thus to their electron transport function, and not due to deformations caused by other stress reactions of the respective cells.

Therefore, the relative intensity (I_Raman_) of the two Raman lines (relRI) could be used as a functional marker for the summarized redox state (ferrous vs. ferric) of all the cytochrome c of the respective bacterial cell, and thus its ongoing electron transport activity at the time of measurement

relRI (cell) = I_Raman_(ν_15_)/I_Raman_(ν_19_), (1)

with I_Raman_(ν) being the intensity of the Raman line associated with the respective vibration (ν) in the spectrum of that individual bacterial cell. 

**Figure 2:**
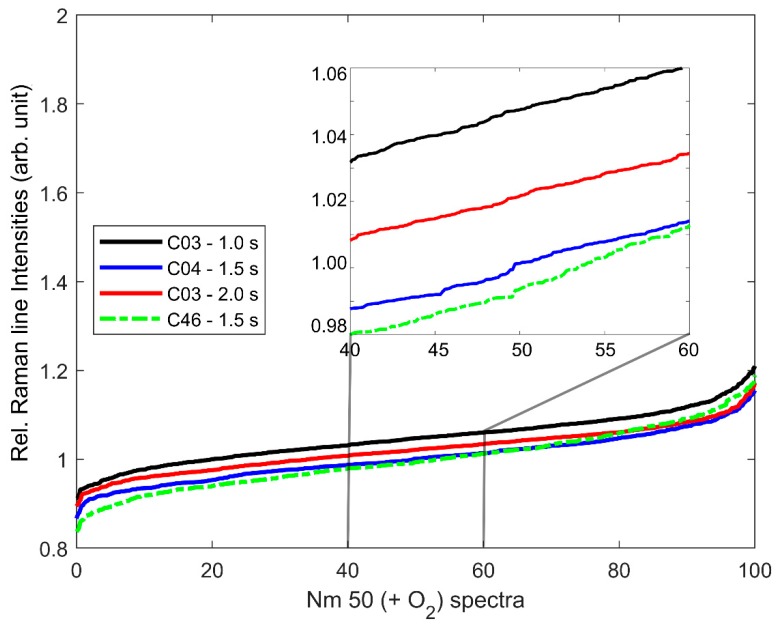
relRI sorted in ascending order of 100 random cells each from four different batches of Nm 50 grown non-simultaneously from two different stock cultures (C03, C46) under optimal growth conditions. The underlying Raman spectra were recorded with three different excitation times (1.0 s, 1.5 s, 2.0 s).

However, since the absolute value of the relRI was dependent on the Raman spectrum of the respective bacteria species (compare Figure 1a,c,e,g), a comparison with the relRI of an unstressed culture of the respective species was required. However, the unstressed reference did not have to be taken from the same culture or even with the same system settings. Figure 2 gives four relRI values determined with three different excitation times (1.0 s, 1.5 s, 2.0 s) for 100 cells each from unstressed *N. europaea* (Nm 50) cultures grown from two different stock cultures (C03, C46) as an example. As can be seen, the differences between the relRI of the respective cultures and measurements were below 0.06, corresponding to 100 ccd counts in the underlying Raman spectra, which was in the order of the random noise in the underlying Raman spectra (compare Figure 1a), despite the Raman spectra recorded from *N. europaea* (Nm 50) having the lowest signal-to-noise ratio (SNR) of all Raman spectra recorded from the analyzed AOB species.

### 3.2. Effects of Oxygen Stress on the Cytochrome-C Content of Nitrosomonas and Nitrosospira

**Figure 3:**
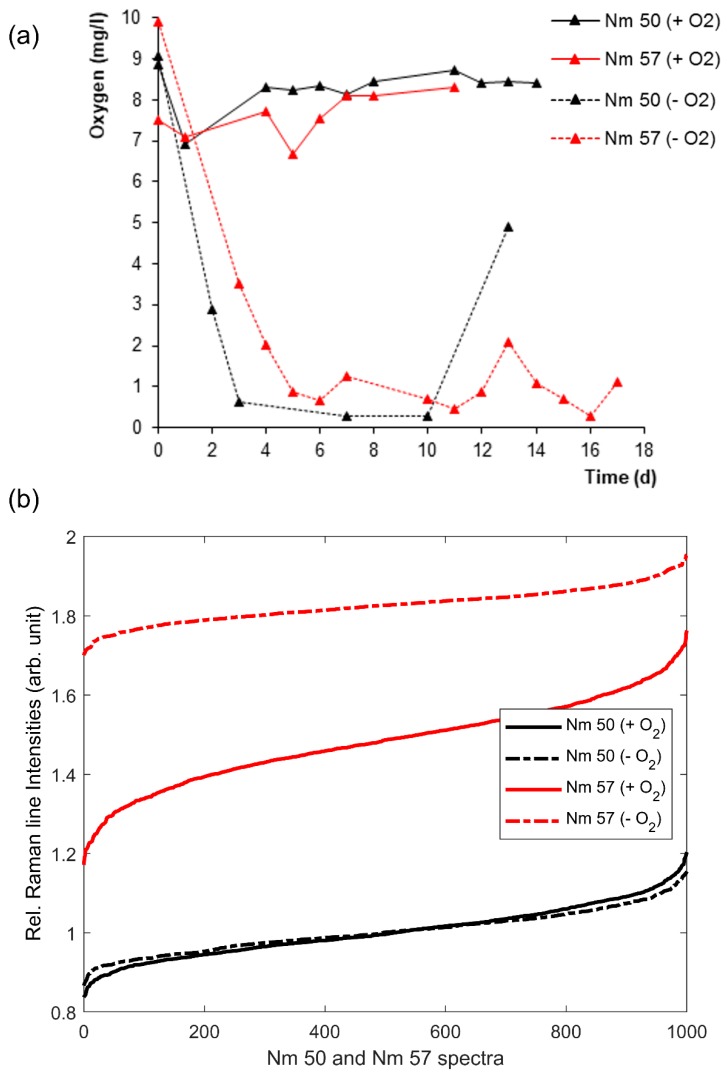
Oxygenation history (**a**) and relative Raman line intensities (relRI) sorted in ascending order of 1000 random cells each (**b**) of *Nitrosomonas europaea* (Nm 50) (black) and *Nitrosomonas eutropha* (Nm 57) (red) grown under well-aerated (solid) or oxygen-deprived (dashed) conditions.

**Figure 4:**
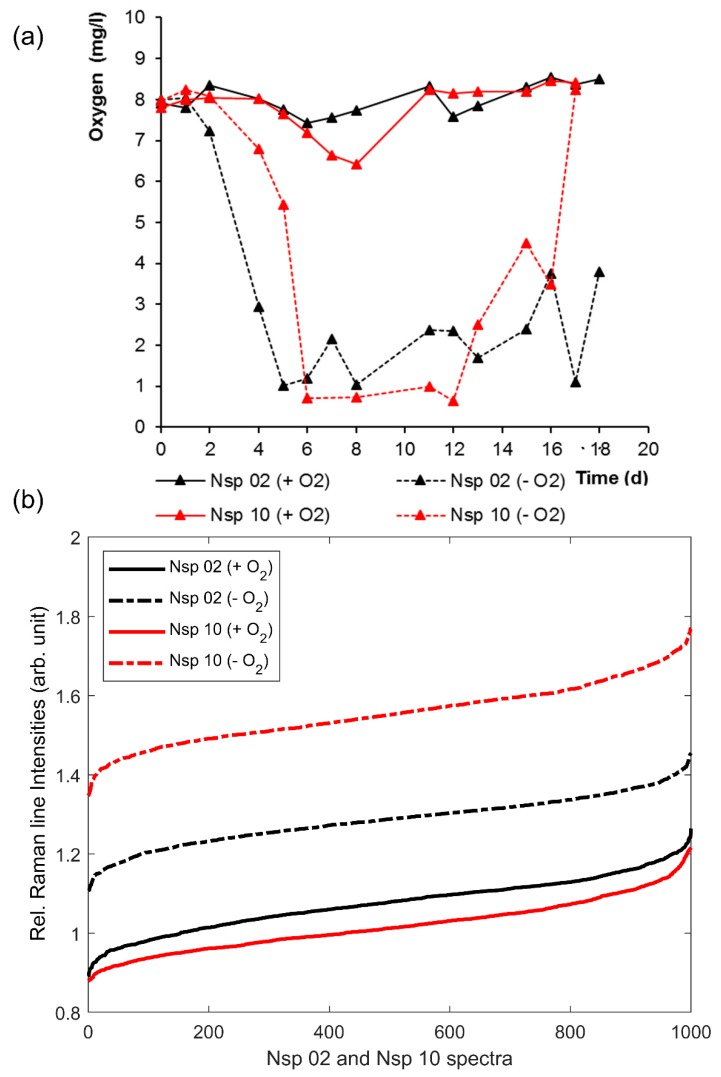
Oxygenation history (**a**) and relative Raman line intensities (relRI) sorted in ascending order of 1000 random cells each (**b**) of *Nitrosospira* sp. (Nsp 02) (black) and *Nitrosospira briensis* (Nsp 10) (red) grown under well-aerated (solid) or oxygen-deprived (dashed) conditions.

The relRI, determined as described in Section 2 and Section 3.1 for 1000 cells each from well-aerated or oxygen-deprived cultures of *N. europaea* (Nm 50) and *N. eutropha* (Nm 57), respectively, and their corresponding oxygenation histories during cultivation are given in Figure 3. As can be seen, the relRI trends of *N. europaea* (black) and *N. eutropha* (red) differed significantly. While the relRI of *N. europaea* was unaffected by the culture conditions, the relRI of oxygen-deprived *N. eutropha* exceeded that of non-deprived *N. eutropha* on average by 0.3, with the non-deprived culture containing a significantly larger percentage of more ferric cells, i.e., cells with a low relRI, than the deprived culture, resulting in a relRI difference ranging from 0.5 for the least ferrous cells in the samples to 0.1 for the most ferrous cells. Moreover, the relRI trends of *Nitrosospira* sp. (Nsp 02) and *Nitrosospira briensis* (Nsp 10) (see Figure 4) showed the same relationship between previous oxygen-deprivation and significantly higher relRI, with the relRI of oxygen-deprived Nsp 02 exceeding that of well-aerated Nsp 02 by 0.2 and Nsp 10 showing a difference 0.4 on average between oxygen-deprived and well-aerated cultures. Given that the Raman spectra underling the relRI trends were recorded from individual cells in a monolayer of cells, this indicated a significantly higher active cytochrome-c content per cell for *N. eutropha* and *Nitrosospira* grown under oxygen-deprived conditions than was seen in cells grown in oxygen abundance.

The different oxygen profiles corresponding to aerated (+O_2_) and oxygen-deprived (−O_2_) culture conditions in Figure 3a and Figure 4a were the result of an interplay of two sequential processes, namely oxygen transfer at the air–liquid interphase and dissolved oxygen consumption in the liquid medium [48]. In the incubation with shaking (+O_2_), the oxygen transfer at the air–liquid interphase was higher compared to the non-shaking condition, which led to a higher oxygen concentration in the medium throughout the entire growth experiment. On the other hand, under a non-shaking condition (−O_2_), oxygen transfer at the air–liquid interphase was slower and, consequently, the oxygen consumption due to ammonium oxidation led to a sharp decrease in the dissolved oxygen concentration. The dissolved oxygen concentration increased towards the end of the growth experiment due to the decrease in ammonium concentration in the medium.

The elevated cytochrome c activity of oxygen-deprived *N. eutropha* and *Nitrosospira* fit well with the results reported by Sedlacek et al. [9] and Pellitteri-Hahn et al. [16], whose works indicated a significant increase in the cytochrome c content of stressed cells, but contradicted Wei et al. [22], who reported mRNA levels for cytochrome c in growing cells to exceed those of energy-starved cells by a factor of 10. Sedlacek et al. [9] used transcriptomics on stressed *N. europaea* to elucidate the stress response to either ammonia or oxygen limitation, reporting that the transcripts for protein-encoding genes related to energy production and conservation, including for the proteins AMO, HAO, and NOR, were increased during oxygen-deprived growth, while three cytochrome c proteins associated with the ammonia-oxidizing pathway were even among the top 20% of genes transcribed under stress conditions. Kozlowski et al. [10], who compared the metabolism pathways of several AOB based on micro-respirometry and genome analysis, confirmed that these enzymes and cytochrome c proteins are encoded in multiple copies by *N. europaea*, *N. eutropha*, and *Nitrosospira*, including the associated cytochrome c proteins.

The observed differences in cytochrome c activity of *Nitrosospira briensis* and *Nitrosospira* sp. (Nsp 02) correlated well with their respective oxygenation histories (compare Figure 4). *Nitrosospira briensis* has been reported to have decreasing AMO mRNA expression during starvation, but to rapidly recover after starvation periods of up to two weeks, with only small changes in the soluble proteins, including cytochrome c, between growing and ammonium-starved cells [7,14]. The oxygen deprivation periods of both cultures, *Nitrosospira briensis* and *Nitrosospira* sp., lasted less than two weeks. While *Nitrosospira briensis* was subjected to a full dissolved oxygen recovery one day before sampling, the oxygen deprivation of the closely related *Nitrosospira* sp. was only reduced by 50%, resulting in a relRI increase of 0.2 for *Nitrosospira* sp. compared to a relRI increase of 0.4 for *Nitrosospira briensis* (see Figure 4).

However, the relRI trends of *N. europaea* did not show an elevated cytochrome c activity indicative of a noticeable stress response in its cells at all (see Figure 3b). We ascribed this to the fact that *N. europaea* appears to be more oxygen-stress-tolerant than either *N. eutropha* or *Nitrosospira* [14,15]. The values of the half oxygen saturation coefficient of *N. europaea* reported in the literature range from 0.22 to 0.56 mg/L [49]. Yu et al. [15], using respirometry-based biokinetic monitoring and real-time reverse-transcriptase-polymerase chain reaction (q-RT-PCR) on *N. europaea* grown under three different oxygen concentrations, reported a significant stress response for *N. europaea* only at the lowest oxygen concentration of 0.5 ± 0.05 mg/L O_2_, a concentration we did not manage to maintain during cultivation (compare Figure 3a). Sedlacek et al. [9] also reported that transient, short-term oxygen deprivation did not lead to a significant stress response in *N. europaea*, leading us to the conclusion that other than with the *N. eutropha* and *Nitrosospira* cultures, we did not manage to induce detectable stress in our *N. europaea* cultures.

Interestingly, the oxygen-deprived *N. eutropha* appeared to have a significantly more evenly distributed cytochrome c activity, with its relRI ranging from 1.7 to 1.9, than non-deprived *N. eutropha*, with a relRI range from 1.2 to 1.75 (see Figure 3). A comparison with the Raman spectra underlying the relRI trends (Figure 1c,d) revealed that this was due to overall more cells, with more ferrous cytochrome c, being present in previously stressed samples compared to unstressed samples. The occurrence of very ferric cells may even have been underreported, given that all four resonantly enhanced Raman lines had to exceed the SNR threshold of two for the spectrum to be admitted to the analysis, including the crucial Raman line at 750 rel. cm^−1^, which is among the weakest lines in ferric spectra, and thus might not always clear the SNR threshold. Moreover, *Nitrosospira* also showed significantly more ferrous cytochrome c content in its oxygen-deprived cultures (Figure 4 and Figure 1e–h). However, other than in *N. eutropha*, cells with more ferric cytochrome c content did occur in oxygen-deprived *Nitrosospira*, resulting in a relRI curve widely parallel to that of the unstressed samples.

The occurrence of cells with extremely ferrous overall cytochrome c content in previously stressed samples may have been caused by electron storage within the ammonia-oxidization pathway that increased the amount of ferrous heme c among the total cytochrome c content of a given cell. A thorough description and overview of this effect studied in detail in *N. europaea* is given in the review of Bewley and Ellis [21]. However, we did not find information about the time scale at which electron storage occurs and is reversed. It can be assumed that the summarized redox state of a given cell changes considerably faster than its total cytochrome c content, which requires protein expression to occur. Therefore, more work is required to elucidate whether the differences in observed extremely ferrous cells in the samples were indeed due to the coping mechanisms for oxygen deprivation differing between *Nitrosomonas* and *Nitrosospira* lineages, as the sampling and preparation steps for microscopy may have affected the electron storage response of *Nitrosomonas* and *Nitrosospira* differently.

## 4. Conclusions

In a proof-of-concept study, confocal Raman microscopy with excitation resonant to the heme c moiety of cytochrome c was used successfully to compare the cytochrome c content and activity of stressed and unstressed *Nitrosomonas europaea* (Nm 50), *Nitrosomonas eutropha* (Nm 57), *Nitrosospira briensis* (Nsp 10), and *Nitrosospira* sp. (Nsp 02) in vivo, based on no more than 1000 individual cells per sampling. This will enable the monitoring of cultures with low cell concentrations before or after sampling for analyses such as proteomics or transcriptomics, which require much higher cell concentrations and considerably larger sample volumes.

The identified spectral marker delivered reproducible results with the given signal-to-noise ratio of the underlying Raman spectra. Cytochrome c content was found to be elevated in oxygen-deprived and previously oxygen-deprived samples. In addition, the marker allowed the distinction between cells with more ferric or more ferrous cytochrome c content, giving insight in the ongoing cytochrome c electron transport activity at the time of measurement. Notably, cells with predominantly ferrous cytochrome c content were found in deprived *N. eutropha* and *Nitrosospira* samples, which may be indicative of ongoing electron storage at the time of measurement. However, more work is needed to confirm whether or not this was due to the previous oxygen-deprivation or constituted a swift reaction to the sampling process. A further study comparing the results obtained with resonant Raman microscopy and quantified results obtained by proteomics or transcriptomics of the same cultures sampled at the same time might allow a quantification of the cytochrome c content elevation reported by Raman microscopy.

## References

[1] Monteiro M., Seneca J., Magalhaes C. (2014). The history of aerobic ammonia oxidizers: From the first discoveries to today. J. Microbiol..

[2] Lehtovirta-Morley L.E. (2018). Ammonia oxidation: Ecology, physiology, biochemistry and why they must all come together. FEMS Microbiol. Lett..

[3] Koops H.P., Purkhold U., Pommerening-Röser A., Timmermann G., Wagner M., Dworking M., Falkow S., Rosenberg E., Schleifer K.H., Stackebrandt E. (2003). The lithoautotrophic ammonia-oxidizing bacteria. The Prokaryotes: An Evoluting Electronic Resource for the Microbiological Community.

[4] Purkhold U., Pommerening-Röser A., Juretschko S., Schmid M.C., Koops H.P., Wagner M. (2000). Phylogeny of all recognized species of ammonia oxidizers based on comparative 16S rRNA and amoA sequence analysis: Implications for molecular diversity surveys. Appl. Environ. Microbiol..

[5] Koops H.-P., Böttcher B., Möller U.C., Pommerening-Röser A., Stehr G. (1991). Classification of eight new species of ammonia-oxidizing bacteria: Nitrosomonas communis sp. nov., Nitrosomonas ureae sp. nov., Nitrosomonas aestuarii sp. nov., Nitrosomonas marina sp. nov., Nitrosomonas nitrosa sp. nov., Nitrosomonas eutropha sp. nov., Nitrosomonas oligotroph asp. Nov. and Nitrosomonas halophila sp. nov. Microbiology.

[6] Koops H.-P., Pommerening-Röser A. (2001). Distribution and ecophysiology of the nitrifying bacteria emphasizing cultured species. FEMS Microbiol. Ecol..

[7] Bollmann A., Bär-Gilissen M.-J., Laanbroek H.J. (2002). Growth at low ammonium concentrations and starvation response as potential factors involved in niche differentiation among ammonia-oxidizing bacteria. Appl. Environ. Microbiol..

[8] Wilhelm R., Abeliovich A., Nejidat A. (1998). Effect of long-term ammonia starvation on the oxidation of ammonia and hydroxylamine by Nitrosomonas europaea. J. Biochem..

[9] Sedlacek C.J., Giguere A.T., Dobie M.D., Mellbye B.L., Ferrell R.V., Woebken D., Sayavedra-Soto L.A., Bottomley P.J., Daims H., Wagner M. (2020). Transcriptomic Response of Nitrosomonas europaea Transitioned from Ammonia- to Oxygen-Limited Steady-State Growth. mSystems.

[10] Kozlowski J.A., Kits K.D., Stein L.Y. (2016). Comparison of Nitrogen Oxide Metabolism among Diverse Ammonia-Oxidizing Bacteria. Front. Microbiol..

[11] Lehnert N., Dong H.T., Harland J.B., Hunt A.P., White C.J. (2018). Reversing nitrogen fixation. Nat. Rev. Chem..

[12] Chu H., Fujii T., Morimoto S., Lin X., Yagi K. (2008). Population size and specific nitrification potential of soil ammonia-oxidizing bacteria under long-term fertilizer management. Soil Biol. Biochem..

[13] Schepers J.S. (2008). Nitrogen in Agricultural Systems.

[14] Geets J., Boon N., Verstraete W. (2006). Strategies of aerobic ammonia-oxidizing bacteria for coping with nutrient and oxygen fluctuations. FEMS Microbiol. Ecol..

[15] Yu R., Chandran K. (2010). Strategies of Nitrosomonas europaea 19718 to counter low dissolved oxygen and high nitrite concentrations. BMC Microbiol..

[16] Pellitteri-Hahn M.C., Halligan B.D., Scalf M., Smith L., Hickey W.J. (2011). Quantitative proteomic analysis of the chemolithoautotrophic bacterium Nitrosomonas europaea: Comparison of growing- and energy-starved cells. J. Proteomics.

[17] Arp D.J., Sayavedra-Soto L.A., Hommes N.G. (2002). Molecular biology and biochemistry of ammonia oxidation by Nitrosomonas europaea. Arch. Microbiol..

[18] Caranto J.D., Vilbert A.C., Lancaster K.M. (2016). Nitrosomonas europaea cytochrome P460 is a direct link between nitrification and nitrous oxide emission. Proc. Natl. Acad. Sci. USA.

[19] Iverson T.M., Arciero D.M., Hooper A.B., Rees D.C. (2001). High-resolution structures of the oxidized and reduced states of cytochrome c554 from Nitrosomonas europaea. J. Biol. Inorg. Chem..

[20] Upadhyay A.K., Hooper A.B., Hendrich M.P. (2006). NO reductase activity of the tetraheme cytochrome C554 of Nitrosomonas europaea. J. Am. Chem. Soc..

[21] Bewley K.D., Ellis K.E., Firer-Sherwood M.A., Elliott S.J. (2013). Multi-heme proteins: nature’s electronic multi-purpose tool. Biochim. Biophys. Acta.

[22] Wei X., Yan T., Hommes N.G., Liu X., Wu L., McAlvin C., Klotz M.G., Sayavedra-Soto L.A., Zhou J., Arp D.J. (2006). Transcript profiles of Nitrosomonas europaea during growth and upon deprivation of ammonia and carbonate. FEMS Microbiol. Lett..

[23] Bollmann A., Schmidt I., Saunders A.M., Nicolaisen M.H. (2005). Influence of starvation on potential ammonia-oxidizing activity and amoA mRNA levels of Nitrosospira briensis. Appl. Environ. Microbiol..

[24] Rygula A., Majzner K., Marzec K.M., Kaczor A., Pilarczyk M., Baranska M. (2013). Raman spectroscopy of proteins: A review. J. Raman Spectrosc..

[25] Kniggendorf A.-K., Schmidt D., Roth B., Plettenburg O., Zeilinger C. (2019). pH-Dependent Conformational Changes of KcsA Tetramer and Monomer Probed by Raman Spectroscopy. Int. J. Mol. Sci..

[26] Kniggendorf A.-K., Meinhardt-Wollweber M., Yuan X., Roth B., Seifert A., Fertig N., Zeilinger C. (2014). Temperature-sensitive gating of hCx26: High-resolution Raman spectroscopy sheds light on conformational changes. Biomed. Opt. Express.

[27] Kniggendorf A.-K., Gaul T.W., Meinhardt-Wollweber M. (2011). Effects of ethanol, formaldehyde, and gentle heat fixation in confocal resonance Raman microscopy of purple nonsulfur bacteria. Microsc. Res. Tech..

[28] Kniggendorf A.-K., Meinhardt-Wollweber M. (2011). Of microparticles and bacteria identification--(resonance) Raman micro-spectroscopy as a tool for biofilm analysis. Water Res..

[29] Dina N.E., Leş A., Baricz A., Szöke-Nagy T., Leopold N., Sârbu C., Banciu H.L. (2017). Discrimination of haloarchaeal genera using Raman spectroscopy and robust methods for multivariate data analysis. J. Raman Spectrosc..

[30] Maquelin K., Dijkshoorn L., van der Reijden T.J.K., Puppels G.J. (2006). Rapid epidemiological analysis of Acinetobacter strains by Raman spectroscopy. J. Microbiol. Methods.

[31] Kniggendorf A.-K., Nogueira R., Kelb C., Schadzek P., Meinhardt-Wollweber M., Ngezahayo A., Roth B. (2016). Confocal Raman microscopy and fluorescent in situ hybridization—A complementary approach for biofilm analysis. Chemosphere.

[32] Kniggendorf A.-K., Gaul T.W., Meinhardt-Wollweber M. (2011). Hierarchical Cluster Analysis (HCA) of Microorganisms: An Assessment of Algorithms for Resonance Raman Spectra. Appl. Spectrosc..

[33] Hu S., Morris I.K., Singh J.P., Smith K.M., Spiro T.G. (1993). Complete assignment of cytochrome c resonance Raman spectra via enzymic reconstitution with isotopically labeled hemes. J. Am. Chem. Soc..

[34] Li X.Y., Czernuszewicz R.S., Kincaid J.R., Stein P., Spiro T.G. (1990). Consistent porphyrin force field. 2. Nickel octaethylporphyrin skeletal and substituent mode assignments from nitrogen-15, meso-d4, and methylene-d16 Raman and infrared isotope shifts. J. Phys. Chem..

[35] Russo V., Candeloro P., Malara N., Perozziello G., Iannone M., Scicchitano M., Mollace R., Musolino V., Gliozzi M., Carresi C. (2019). Key Role of Cytochrome C for Apoptosis Detection Using Raman Microimaging in an Animal Model of Brain Ischemia with Insulin Treatment. Appl. Spectrosc..

[36] Kakita M., Kaliaperumal V., Hamaguchi H. (2012). Resonance Raman quantification of the redox state of cytochromes b and c in-vivo and in-vitro. J. Biophotonics.

[37] Shin H.J., Lee J.H., Kim Y.D., Shin I., Sim T., Lim D.-K. (2019). Raman-Based in Situ Monitoring of Changes in Molecular Signatures during Mitochondrially Mediated Apoptosis. ACS Omega.

[38] Zhang J., Ma X., Wang Z. (2019). Surface-Enhanced Raman Scattering-Fluorescence Dual-Mode Nanosensors for Quantitative Detection of Cytochrome c in Living Cells. Anal. Chem..

[39] Zhu J., Jiang M., Ma H., Zhang H., Cheng W., Li J., Cai L., Han X.X., Zhao B. (2019). Redox-State-Mediated Regulation of Cytochrome c Release in Apoptosis Revealed by Surface-Enhanced Raman Scattering on Nickel Substrates. Angew. Chem. Int. Ed. Engl..

[40] Krümmel A., Harms H. (1982). Effect of organic matter on growth and cell yield of ammonia-oxidizing bacteria. Arch. Microbiol..

[41] Bremner J.M., Keeney D.R. (1965). Steam distillation methods for determination of ammonium, nitrate and nitrite. Anal. Chim. Acta.

[42] Koch M., Suhr C., Roth B., Meinhardt-Wollweber M. (2017). Iterative morphological and mollifier-based baseline correction for Raman spectra. J. Raman Spectrosc..

[43] Koch M., Kniggendorf A.-K., Meinhardt-Wollweber M., Roth B. (2018). In vivo determination of carotenoid resonance excitation profiles of Chlorella vulgaris, Haematococcus pluvialis, and Porphyridium purpureum. J. Raman Spectrosc..

[44] Koch M., Zagermann S., Kniggendorf A.-K., Meinhardt-Wollweber M., Roth B. (2017). Violaxanthin cycle kinetics analysed in vivo with resonance Raman spectroscopy. J. Raman Spectrosc..

[45] The Mathworks Inc. (2019). MATLAB (Version 2019a Update 6). Windows.

[46] Murgida D.H., Hildebrandt P. (2004). Electron-transfer processes of cytochrome C at interfaces. New insights by surface-enhanced resonance Raman spectroscopy. Acc. Chem. Res..

[47] Murgida D.H., Hildebrandt P. (2008). Disentangling interfacial redox processes of proteins by SERR spectroscopy. Chem. Soc. Rev..

[48] Maier U., Büchs J. (2001). Characterisation of the gas-liquid mass transfer in shaking bioreactors. Biochem. Eng. J..

[49] Laanbroek H.J., Bodelier P.L.E., Gerards S. (1994). Oxygen consumption kinetics of Nitrosomonas europaea and Nitrobacter hamburgensis grown in mixed continuous cultures at different oxygen concentrations. Arch. Microbiol..

